# Neuroanatomical characterization of the cell adhesion molecule IgSF9b reveals localization to inhibitory and excitatory synapses in the mouse limbic system

**DOI:** 10.1007/s00109-025-02600-6

**Published:** 2025-10-17

**Authors:** Federico Rotondo, Heba Ali, Maxim Maichle, Michael J. Schmeisser, Nils Brose, Dilja Krueger-Burg

**Affiliations:** 1 Institute of Anatomy, University Medical Center of the Johannes Gutenberg University Mainz, Duesbergweg 6, 55128 Mainz, Germany; 2 Department of Molecular Neurobiology, Max Planck Institute of Multidisciplinary Sciences, Hermann-Rein-Str. 3, 37075 Göttingen, Germany; 3 Focus Program Translational Neurosciences, University Medical Center of the Johannes Gutenberg University Mainz, Duesbergweg 6, 55128 Mainz, Germany

**Keywords:** GABA, Inhibition, Interneuron, Hippocampus, Amygdala

## Abstract

**Abstract:**

Immunoglobulin superfamily member 9b (IgSF9b) is a cell adhesion protein that has been linked to the etiology of several neuropsychiatric disorders, most notably schizophrenia and major depression. Based on previous studies in cultures, IgSF9b was proposed to specifically regulate the structure and function of inhibitory synapses in the brain through an indirect interaction with the synaptic adhesion protein Neuroligin-2 (Nlgn2). However, very little is known about the protein expression pattern of IgSF9b in the intact brain, and the synaptic localization of IgSF9b in different brain regions has never been investigated. To address this question, we conducted an immunohistochemical characterization of IgSF9b expression across the mouse brain and investigated its colocalization with gephyrin, Nlgn2 and VIAAT as markers of GABAergic inhibitory synapses, as well as with PSD-95 and VGLUT1 as markers of glutamatergic excitatory synapses. Unexpectedly, we observed that in the brain regions assessed, only a small fraction of IgSF9b puncta colocalized with inhibitory marker puncta, with a similarly small fraction colocalizing with excitatory synapses. The majority of IgSF9b puncta were not associated with any of the investigated synaptic markers, indicating that IgSF9b may have additional functions beyond those at GABAergic and glutamatergic synapses. Moreover, deletion of IgSF9b resulted in alterations in inhibitory synapse markers in the stratum lacunosum moleculare of hippocampal area CA1 as well as in the lateral and medial habenula, which play key roles in the regulation of cognitive and affective behaviors, respectively. Together, our findings provide an important context for the assessment of the role of IgSF9b in neuropsychiatric disorders.

**Key messages:**

- IgSF9b is expressed in psychiatrically relevant brain regions in the mouse brain.
- Loss of IgSF9b leads to a small but significant reduction in inhibitory synapses.
- Only a subset of inhibitory synapses in the regions assessed contain IgSF9b.
- IgSF9b is additionally present at a subset of excitatory synapses in these regions.
- Only 20% of IgSF9b is localized to synapses, while the majority is non-synaptic.

**Supplementary Information:**

The online version contains supplementary material available at 10.1007/s00109-025-02600-6.

## Introduction

Disruptions in the synaptic communication between neurons in the brain are increasingly emerging as a common pathophysiological pathway in neuropsychiatric disorders [1–3]. In particular, alterations in the balance between glutamatergic excitatory synaptic transmission and γ-aminobutyric acid (GABA)-ergic inhibitory synaptic transmission have been postulated as a central mechanism, resulting in aberrant neuronal information processing and deficits in behavioral and cognitive functions [4, 5]. While the majority of these studies have focused on the role of excitatory synapses in neuropsychiatric pathophysiology, a growing body of evidence indicates that alterations at GABAergic inhibitory synapses also contribute to the development of these disorders [6–8]. Accordingly, targeting GABAergic synaptic transmission represents a highly interesting strategy for the development of new therapeutic approaches in psychiatry [9–11].

In addition to ligand-gated GABA_A_ receptors (GABA_A_Rs), GABAergic synapses contain synaptic organizer proteins that regulate their formation, functional properties, and plasticity, including a large number of cell adhesion proteins that mediate the interaction between the pre- and postsynaptic compartments [12–14]. Recent evidence indicates that these proteins differentially regulate synapses formed by different inhibitory neuron subtypes, contributing to the molecular diversity of GABAergic synaptic transmission [14, 15]. Given that several of these organizer proteins have been linked to the etiology of neuropsychiatric disorders [16], understanding how these proteins contribute to the structure and function of different synapse subtypes is essential for evaluating their potential as targets for therapeutic strategies [15].

One of these proposed organizer proteins is the cell adhesion protein Immunoglobulin superfamily member 9b (IgSF9b) [17, 18]. In genome-wide association analyses, the IGSF9B locus was found to be linked to the etiology of schizophrenia [19–24] and major depression [25], as well as to Parkinson’s disease [24, 26, 27], multiple sclerosis [28, 29], and migraine [30]. At the synaptic level, IgSF9b was initially reported to be localized specifically to inhibitory, but not excitatory, synapses in dissociated hippocampal cultures, where it was proposed to form a complex with the inhibitory synaptic adhesion protein Neuroligin-2 (Nlgn2) and the scaffolding protein S-SCAM [31]. Moreover, knockdown of IgSF9b levels was found to result in a modest loss of GABAergic synapses specifically from interneurons, leading the authors to conclude that IgSF9b promotes inhibitory synapse development. However, subsequent studies in intact mouse brains painted a more differentiated picture, with loss of IgSF9b expression leading to an increase rather than, or in addition to, a decrease in certain GABAergic synapse markers in the centromedial amygdala [32] and visual cortex [33]. These findings indicate that IgSF9b may regulate GABAergic synaptic function in a brain region- and/or synapse subtype-dependent manner, but to date, no detailed information on the region-specific protein expression pattern and synaptic localization of IgSF9b is available.

In the present study, we address this issue by performing a neuroanatomical characterization of IgSF9b expression, as well as investigating the colocalization of IgSF9b with synaptic markers and determining the consequences of IgSF9b deletion on GABAergic synapses in a subset of relevant regions relevant for neuropsychiatric disorders, including the hippocampus, basolateral amygdala, and medial and lateral habenula. Together, our data provide an important basis for understanding the molecular mechanisms linking IgSF9b dysfunction to neuropsychiatric pathophysiology and treatment.

## Results

### IgSF9b is highly expressed in regions involved in cognitive, motor and affective behaviors

**Fig. 1:**
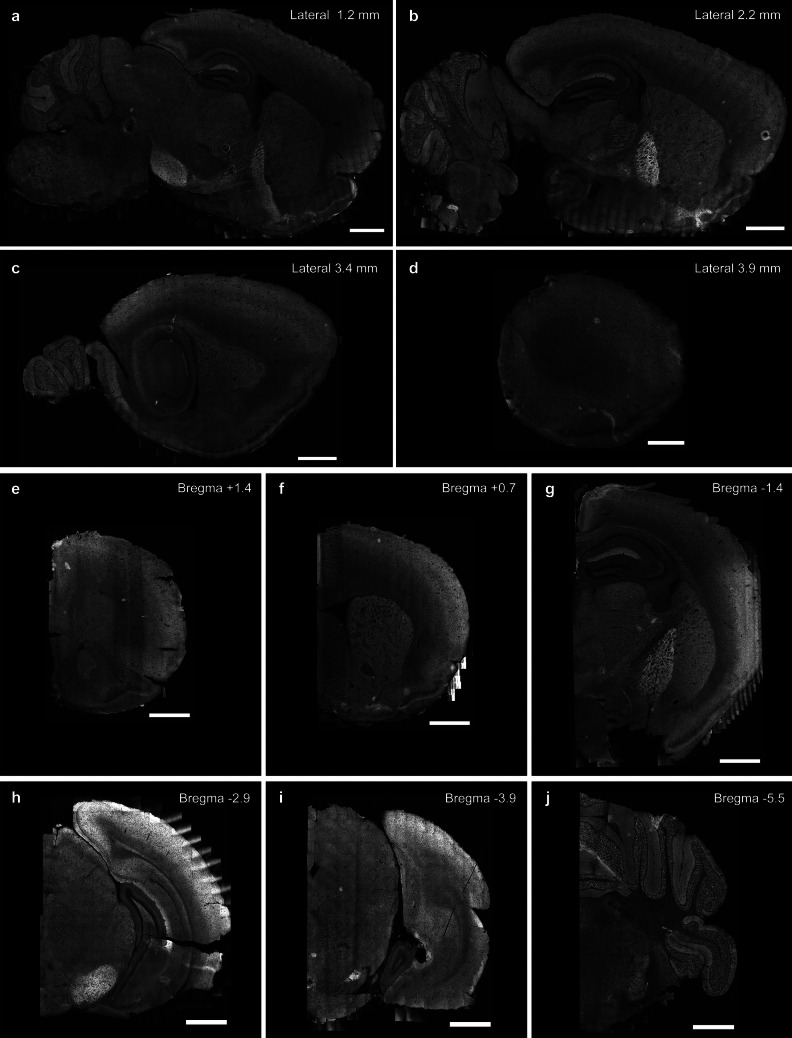
Neuroanatomical characterization of IgSF9b expression pattern. **a-d** Sagittal sections at + 1.2 mm **a**, + 2.2 mm **b**, + 3.4 mm **c** and + 3.9 mm **d** lateral to the midline. **e-j** Coronal sections at Bregma + 1.4 mm **e**, + 0.7 mm **f**, −1.4 mm g, −2.9 mm h, −3.9 mm **i** and −5.5 mm **j**. Scale bar: 1 mm

**Fig. 2:**
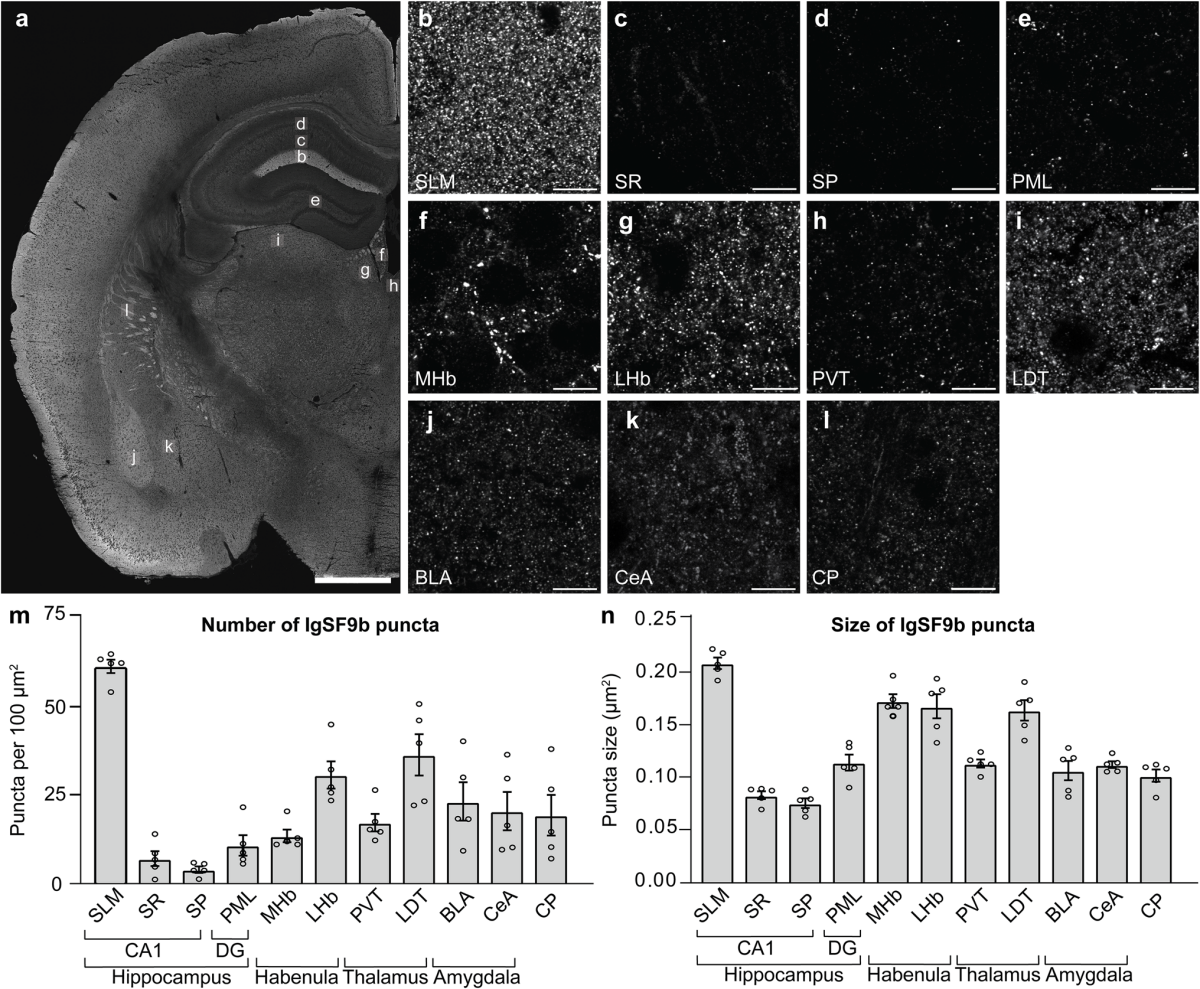
IgSF9b is highly expressed in brain regions involved in cognitive and affective disorders. **a** Low magnification photomicrograph showing immunostaining for IgSF9b in a coronal brain section (see also Figure S1 for IgSF9b KO control). Letters denote location of high magnification photomicrographs displayed in (b-l). **b-l** High magnification photomicrographs showing immunostaining for IgSF9b in the SLM **b**, SR **c**, and SP **d** of hippocampal area CA1, PML of the dentate gyrus **e**, MHb (f), LHb **g**, PVT **h**, LDT **i**, BLA **j**, CeA **k** and CP **l**. **m–n** Quantification of the number **m** and size **n** of IgSF9b puncta in non-cortical regions in WT mice (*n* = 5). Error bars represent SEM. Abbreviations. BLA, basolateral amygdala; CA1, area cornu ammonis 1 of the hippocampus; CP, caudate/putamen; CeA, central amygdala. DG, dentate gyrus of the hippocampus; LDT: laterodorsal nucleus of the thalamus; PVT, paraventricular nucleus of the thalamus; LHb, lateral habenula; MHb, medial habenula; PML, polymorphic layer; SP, stratum pyramidale; SR, stratum radiatum; SLM, stratum lacunosum moleculare. Scale bars: **a** 1 mm, **b** –**l** 10 µm

While previous studies have reported brain-wide analyses of IgSF9b mRNA expression patterns [17, 31], no equivalent information is available for IgSF9b protein localization patterns. Given that the cellular localization of synaptic proteins often does not correspond to their transcriptional patterns, we sought to address this issue by performing a brain-wide immunohistochemical analysis on sagittal (Fig. 1a-d) and coronal (Fig. 1e-j, Fig. 2a) sections of adult WT mouse brain. To this end, we first re-confirmed the specificity of an IgSF9b antibody that we had used and validated by immunoblotting and immunohistochemistry in a prior study [32] under our current experimental conditions (Figure S1a-d). Subsequent immunohistochemical analysis revealed a clear pattern of IgSF9b expression throughout the brain, including in areas that were the focus of previous mRNA expression analyses, such as the cortex and cerebellum [17, 31]. In addition, a particularly striking labeling was observed in hippocampal area CA1, and specifically in the stratum lacunosum moleculare (SLM) (Fig. 1a, b, g), which receives inputs from the entorhinal cortex via the perforant pathway and plays a key role in cognitive processing. Strong expression was also observed in the globus pallidus and substantia nigra, both of which are involved prominently in motor control (Fig. 1a, b, g, h). Further regions of high expression included the medial habenula (MHb), lateral habenula (LHb), basolateral amygdala (BLA), as well as the lateral dorsal nucleus of the thalamus (LDT) and central amygdala (Fig. 1g, Fig. 2a), all of which connect to limbic regions and mediate emotional processing [34, 35].

High magnification images of some of the most highly IgSF9b-expressing regions revealed that IgSF9b shows a discrete punctate staining pattern reminiscent of synaptic staining, consistent with the current notion that IgSF9b is localized synaptically (Fig. 2b-l). A notable exception appears to be found in the cerebellum, where intense labeling of large clusters of IgSF9b puncta was observed throughout the granular layer (Figure S2a), precluding quantification of puncta using the same parameters as for the other regions. Nevertheless, total staining intensity in the cerebellar granular layer approximately equaled that in layer SLM in hippocampal area CA1 (Figure S2b-d), showing strong correlation with the mRNA expression pattern reported in the Allen Brain Atlas ISH data set (Experiment 75147761).

Given that substantial interest in the clinical relevance of IgSF9b has focused on its link to schizophrenia and major depression [17, 20, 21], we decided to focus our further investigations on regions known to contribute to encoding cognitive and affective behaviors, including SLM of hippocampal area CA1, MHb, LHb, and BLA.

### Global deletion of IgSF9b leads to reductions in the number and size of inhibitory synapses in a region-dependent manner

**Fig. 3:**
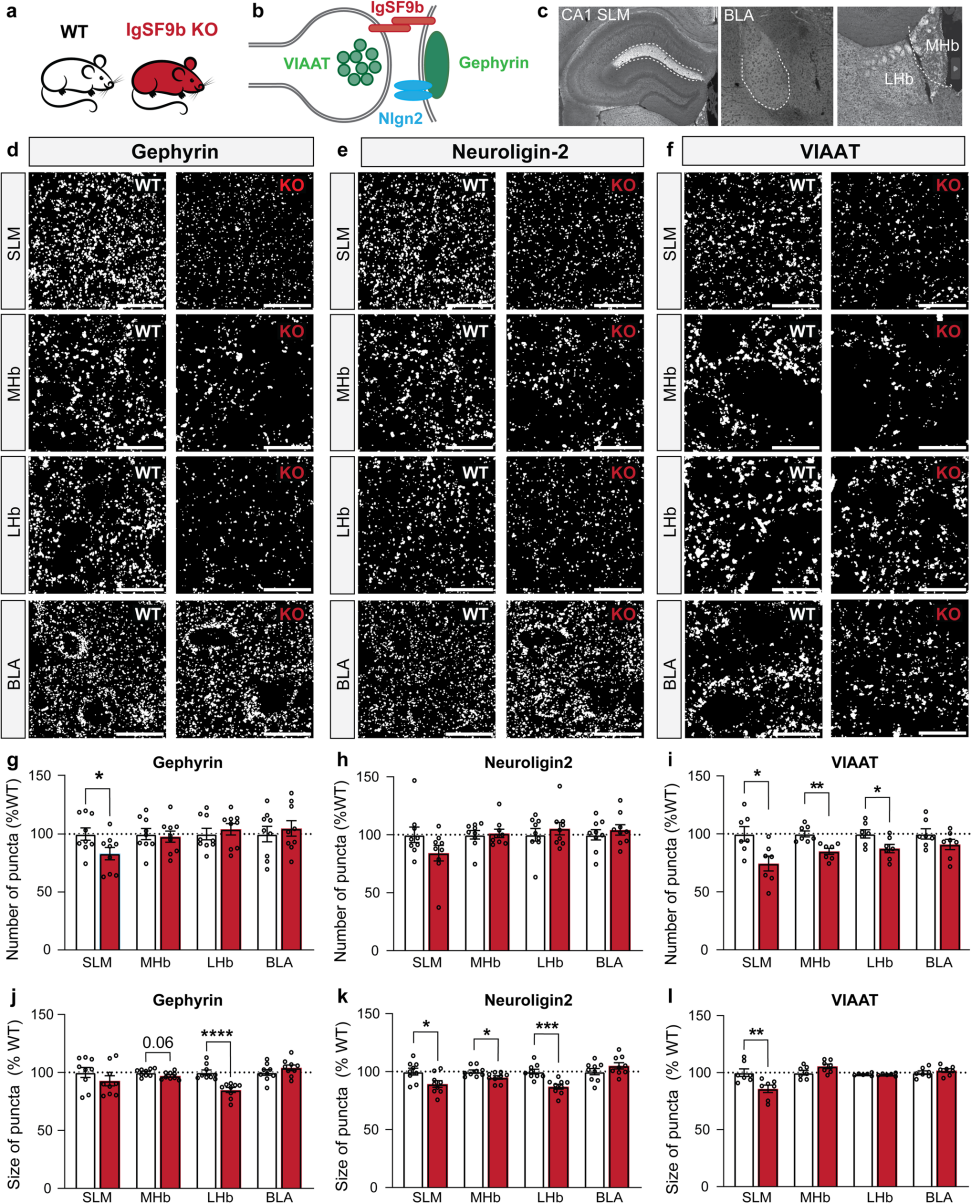
Loss of IgSF9b expression leads to alterations of inhibitory markers. **a** Schematic representation of genotypes assessed, i.e., IgSF9b WT and KO mice. **b** Schematic representation of localization of synaptic markers. **c** Anatomical representation of the brain regions investigated. **d-f** Photomicrographs of gephyrin **d**, Nlgn2 **e**, and VIAAT **f** immunostaining in the SLM, MHb, LHb, and BLA of IgSF9b WT and KO mice (*n* = 7). **g-i** Quantification of the number of gephyrin **g**, Nlgn2 **h**, and VIAAT **i** puncta in the SLM, MHb, LHb, BLA of IgSF9b WT and KO mice (white and red bars, respectively; *n* = 7). **j-l** Quantification of the average size of gephyrin **j**, Nlgn2 **k**, and VIAAT **l** puncta in the SLM, MHb, LHb, BLA of IgSF9b WT and KO mice (white and red bars, respectively; *n* = 7). Statistically significant unpaired t-test comparisons are marked with * *p* < 0.05, ** *p* < 0.01, *** *p* < 0.001,**** *p* < 0.0001 and listed in Table S1. Error bars represent SEM. Scale bar: 15 µm

IgSF9b has been proposed to interact with the GABAergic synaptic proteins gephyrin and Nlgn2, and global deletion of IgSF9b in mice has been reported to lead to alterations at GABAergic synapses in the centromedial amygdala [32] and the visual cortex [33]. Accordingly, we asked whether the same deletion also affects GABAergic synapses in the CA1 SLM, LHb, MHb and BLA (where no differences had previously been reported [32]). To this end, we performed immunohistochemical analysis for gephyrin, Nlgn2 and the GABAergic presynaptic marker VIAAT (vesicular inhibitory amino acid transporter) in WT and IgSF9b knockout (KO) mice in the overmentioned brain regions (Fig. 3a-f). As expected, the most consistent alterations were found in the region with the most prominent IgFS9b expression, i.e. the SLM of hippocampal area CA1, where small but significant reductions in the number of gephyrin and VIAAT puncta (16.8% and 25.5% reduction relative to WT control, respectively) as well as in the size of Nlgn2 and VIAAT puncta (10.6% and 14.1% reduction relative to WT control, respectively) were observed (Fig. 3g-l). More modest effects were observed in LHb and MHb, where reductions in the size of gephyrin puncta (15.1% in the LHb) and Nlgn2 puncta (12.9% in the LHb and 5% in the MHb) as well as in the number of VIAAT puncta (12.6% in the LHb and 15% in the MHb) were identified (Fig. 3g-l). In contrast, despite the clear expression of IgSF9b in BLA, no effects on GABAergic synapse markers were identified in this region, consistent with our previous findings [32]. Our results indicate that IgSF9b contributes to the regulation of inhibitory synapse number and/or composition in a brain region-specific manner, including in regions associated with neuropsychiatric disorders.

### Only a small percentage of inhibitory synapses contain IgSF9b

**Fig. 4:**
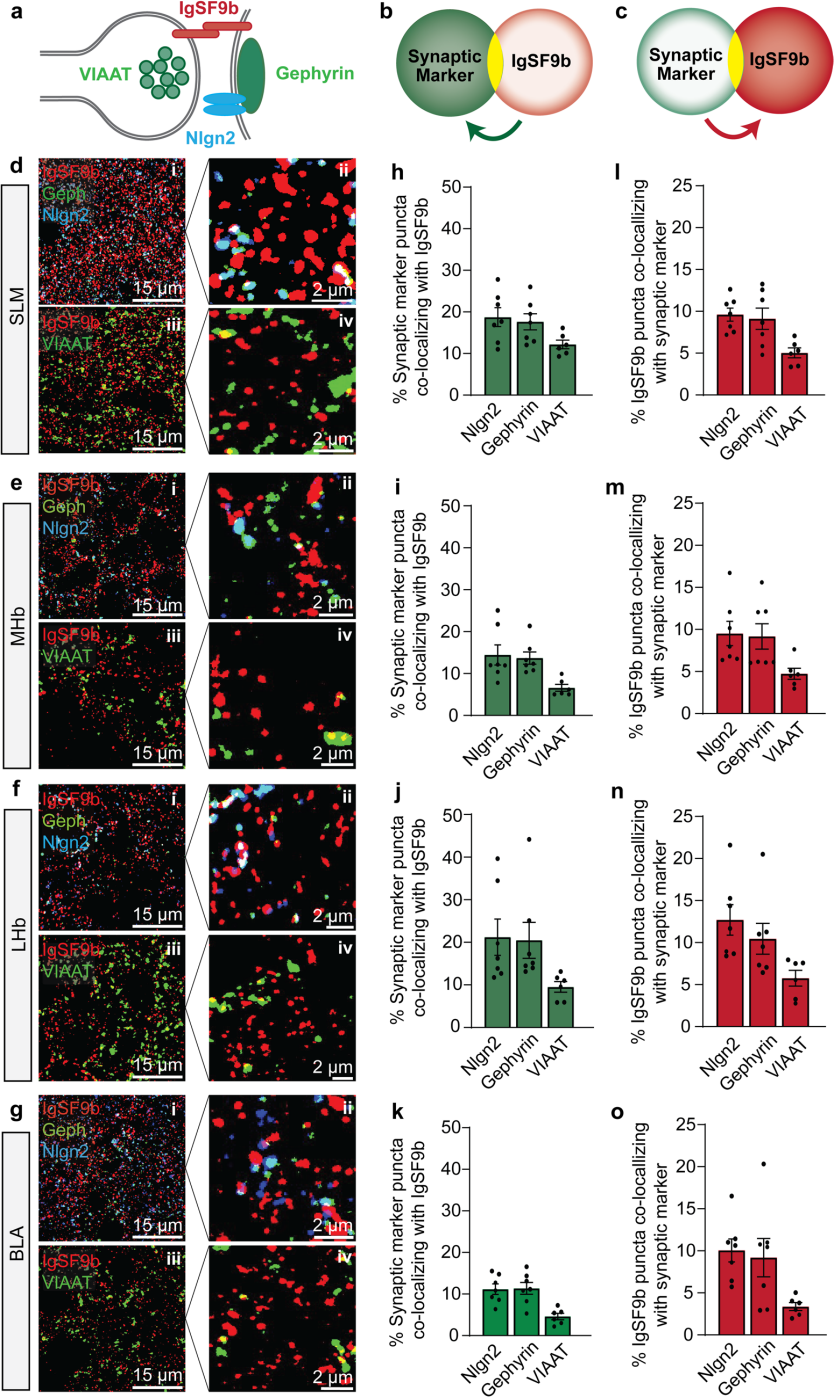
Localization of IgSF9b at inhibitory synapses in different brain regions. **a** Schematic representation of the distribution of synaptic markers at inhibitory synapses. **b** Model of colocalization analysis between synaptic markers and IgSF9b. **c** Model of colocalization analysis between IgSF9b and synaptic markers. **d-g** Representative photomicrographs of colocalization between IgSF9b and inhibitory synapse markers (gephyrin, Nlgn2, and VIAAT) in the SLM **d**, MHb **e**, LHb **f**, and BLA **g**, with each panel containing low and high magnification images of the colocalization of IgSF9b with gephyrin and Nlgn2 in the top row (subpanels i and ii, respectively) and with VIAAT in the bottom row (subpanels iii and iv, respectively). **h–k** Quantification of the percentage of inhibitory synapse marker puncta colocalizing with IgSF9b in SLM **h**, MHb **i**, LHb **j**, and BLA **k**. **l-o** Quantification of the percentage of IgSF9b puncta colocalizing with inhibitory synaptic markers in the SLM **l**, MHb **m**, LHb **n** and BLA **o**. Error bars represent SEM

While we did observe significant alterations at GABAergic synapses in IgSF9b KO mice, they were surprisingly modest in magnitude and restricted to a subset of brain regions and markers. This led us to ask whether IgSF9b is indeed present at all GABAergic synapses in the regions investigated, or whether only a subset of GABAergic synapses contain IgSF9b, potentially indicating a molecular diversity that may explain the relatively small number of GABAergic synapses affected by the loss of IgSF9b expression. To this end, we co-stained for IgSF9b and the inhibitory synapse markers gephyrin, Nlgn2, and VIAAT in the four brain regions assessed above and analyzed the percentage of puncta colocalization (Fig. 4a-g; see also Figure S4a-d for Pearson correlation coefficient). Strikingly, we found that only approximately 10–20% of gephyrin and Nlgn2 puncta also contained IgSF9b expression (Fig. 4h-k). Moreover, this percentage varied by brain region, with the highest percentage in LHb (20.9%% for gephyrin and 21.7% for Nlgn2), followed by CA1 SLM (17.6% for gephyrin and 18.7% for Nlgn2), MHb (13.9% for gephyrin and 14.6% for Nlgn2), and BLA (11.4% for gephyrin and 11.2% for Nlgn2). An even lower percentage of colocalization was observed for the presynaptic inhibitory synapse marker VIAAT, with only 5–12% of VIAAT puncta containing IgSF9b staining (SLM, 12.2%, LHb 9.7%, MHb, 6.7%, BLA, 4.6%). These findings indicate that IgSF9b is not universally present at all GABAergic synapses, but is only indeed found at a small and as yet undefined subset of synapses, and that this subset may be differentially distributed in different brain regions.

### Only a small percentage of IgSF9b is localized to inhibitory synapses

Perhaps even more surprisingly, our colocalization analysis revealed that, not only do most gephyrin, Nlgn2 and VIAAT puncta not contain any detectable IgSF9b, but conversely most IgSF9b puncta are not colocalized with any GABAergic synaptic markers (Fig. 4a-g). Indeed, quantitative analysis of these data reveal that only approximately 10% of IgSF9b puncta overlap with gephyrin and Nlgn2, and only 5% with VIAAT (Fig. 4l-o). Interestingly, unlike the number of GABAergic synapses containing IgSF9b, the percentage of IgSF9b puncta overlapping with gephyrin, Nlgn2 and VIAAT does not seem to vary substantially between brain regions (overlap with gephyrin: SLM, 9.1%; LHb, 10.5%; MHb, 9.1%; BLA, 9.1%; overlap with Nlgn2: SLM, 9.6%; LHb, 12.7%; MHb, 9.5%; BLA, 9.5%; overlap with VIAAT: SLM, 5.0%; LHb, 5.8%; MHb, 4.7%; BLA; 3.3%), indicating that the fraction of IgSF9b localized to GABAergic synapses may not be dependent on the local cellular or synaptic context.

### A small percentage of IgSF9b is localized to excitatory synapses

**Fig. 5:**
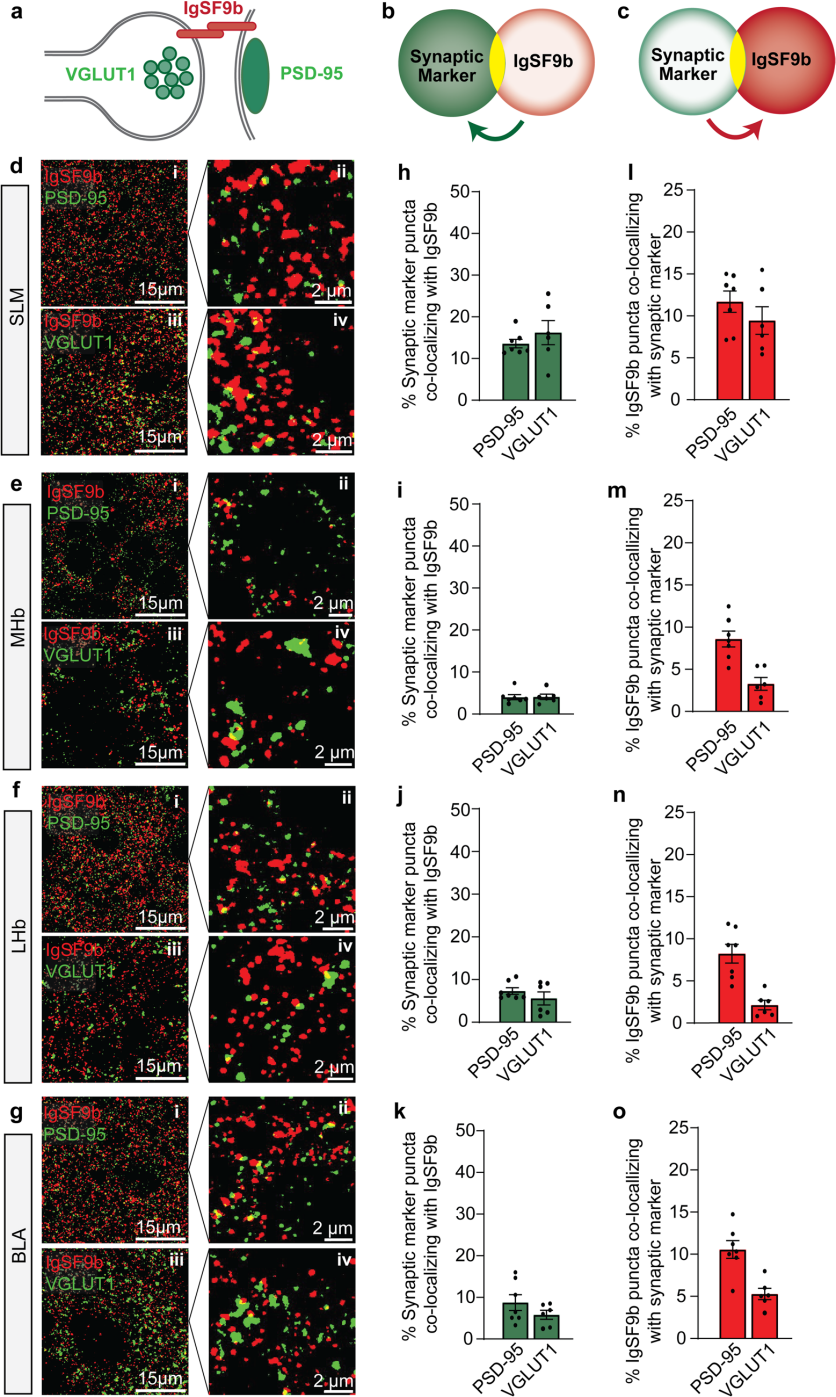
Localization of IgSF9b at excitatory synapses in different brain regions. **a** Schematic representation of the distribution of synaptic markers at excitatory synapses. **b** Model of colocalization analysis between synaptic markers and IgSF9b. **c** Model of colocalization analysis between IgSF9b and synaptic markers. **d** Representative photomicropgrahs of colocalization between IgSF9b and excitatory synapse markers (PSD-95 and VGLUT1) in the SLM **d**, MHb **e**, LHb **f**, and BLA **g**, with each panel containing low and high magnification images of the colocalization of IgSF9b with PSD-95 in the top row (subpanels i and ii, respectively) and with VGLUT1 in the bottom row (subpanels iii and iv, respectively). **h–k** Quantification of the percentage of excitatory synapse marker puncta colocalizing with IgSF9b in SLM **h**, MHb **i**, LHb **j**, and BLA **k**. **l-o** Quantification of the percentage of IgSF9b puncta colocalizing with excitatory synaptic markers in the SLM **l**, MHb **m**, LHb **n** and BLA **o**. Error bars represent SEM

The observation that only about 10% of IgSF9b puncta are colocalized with GABAergic synapse markers of course raises the question of which subcellular structures the remaining 90% of IgSF9b are localized to. One possibility would be that, unlike previously reported for hippocampal cultures, IgSF9b is also present at excitatory, glutamatergic synapses in the adult brain. To address this question, we performed co-staining of IgSF9b with markers of glutamatergic synapses, i.e. PSD-95 and VGLUT1 (Fig. 5a-g, see also Figure S4a-d for Pearson correlation coefficient). This analysis showed that a small fraction of PSD-95 and VGLUT1 puncta do indeed contain IgSF9b, particularly in CA1 SLM (13.6% and 16.2%, respectively) and BLA (8.8% and 5.8%, respectively), but also in LHb (7.5% and 5.7%, respectively) and, to a lesser degree, in MHb (4% and 4.1%, respectively) (Fig. 5h-k). Importantly, in CA1 SLM and BLA, the percentage of PSD-95 and VGLUT1 puncta containing IgSF9b is similar to that of gephyrin and VIAAT puncta containing IgSF9b, respectively, indicating that IgSF9b is not, as previously proposed, specifically localized to GABAergic synapses, at least in certain regions of the adult mouse forebrain. However, as for GABAergic synapse markers, only about 10% of total IgSF9b puncta were found to colocalize with glutamatergic synapse markers (overlap with PSD-95: SLM; 11.7%; LHb; 8.2%; MHb; 8.5%; BLA, 10.6%; overlap with VGLUT1: SLM, 9.4%; LHb, 2.2%; MHb, 3.2%; BLA, 5.2%) (Fig. 5l-o), leaving around 80% of IgSF9b puncta that appear to be localized neither at GABAergic nor at glutamatergic synapses. Determining the localization and function of this substantial fraction of IgSF9b puncta will be key in understanding the full range of mechanisms by which IgSF9b contributes to the etiology of neuropsychiatric disorders.

## Discussion

In the present study, we provide a neuroanatomical characterization of the expression pattern and synaptic localization of the cell adhesion protein IgSF9b, which has been proposed to contribute to the etiology of schizophrenia and other brain disorders through its role at GABAergic synapses. We report that IgSF9b in mice is particularly highly expressed in brain regions associated with cognitive and emotional behaviors, including the hippocampus, lateral and medial habenula, and basal amygdala. Strikingly, however, only approximately 10% of IgSF9b puncta were associated with GABAergic synapses in these regions, with another 10% of puncta localized to glutamatergic synapses, and the vast majority not associated with either synapse subtype. Conversely, only approximately 10–20% of GABAergic synapses contained IgSF9b, and deletion of IgSF9b resulted in loss of only a fraction of GABAergic synapse markers. Our findings indicate that IgSF9b plays a role at a small subset of GABAergic synapses in psychiatrically relevant brain regions, and that it may contribute to the etiology of schizophrenia and other brain disorders both through its function at these synapses and through other, as yet undefined mechanisms of action.

Arguably the most important result from our study is the finding that IgSF9b is not a GABAergic synapse-specific cell adhesion molecule as previously proposed [12, 17, 31], at least not in the adult mouse forebrain. Our data show that, in those forebrain regions where IgSF9b is most highly expressed, only around 10% of IgSF9b puncta colocalize with GABAergic synapse markers, with a further 5–10% colocalizing with glutamatergic synapse markers and the remaining 80% localized to as yet unidentified subcellular locations. These results differ from previous findings in dissociated hippocampal cultures, where a specific colocalization of IgSF9b was observed with gephyrin and the presynaptic marker GAD65, but not with PSD-95 [31]. In this study, it was found that while IgSF9b puncta could be found in close proximity to gephyrin puncta, the direct overlap between the puncta as assessed by confocal microscopy was minimal. Based on super-resolution imaging to provide a more precise spatial assessment, it was proposed that IgSF9b and gephyrin may localize to separate subsynaptic domains at inhibitory synapses [31]. However, no quantification of the degree of colocalization was available in this study, either for GABAergic or for glutamatergic synapse markers, and therefore the extent to which these findings apply to all GABAergic and glutamatergic synapses in hippocampal cultures is unknown. Moreover, at the time, no immunohistochemistry protocols were available for assessment of IgSF9b in the intact adult brain, precluding a neuroanatomical characterization. In a subsequent study, our group developed such a protocol and reported that IgSF9b and Nlgn2 can be found in close proximity in the centromedial amygdala, but not the basal amygdala, of adult mice [32]. Here we show that in the SLM of hippocampal area CA1, the LHb, the MHb and the BLA, only a minor subset of IgSF9b puncta are localized in close proximity to GABAergic synapse markers, with another minor subset localized to glutamatergic synapse markers, and the majority not found overlapping with or in close proximity to any synapse markers that we assessed. It is conceivable that the degree to which IgSF9b localizes to GABAergic and glutamatergic synapses depends on the developmental timepoint. IgSF9b is known to be expressed differentially throughout development [17, 33], and may play a greater role at GABAergic synapses during early postnatal development, i.e. the timepoint assessed in Woo et al. 2013, than in the adult mouse brain as assessed in the present study. Interestingly, it was previously reported that a subset of glutamatergic neurons in the adult mouse cortex express substantially higher levels of IgSF9b than other glutamatergic neurons [17], raising the possibility that these differences in expression pattern may correlate with the differential utilization of IgSF9b at glutamatergic vs. GABAergic synapses.

The nature of the 80% of IgSF9b puncta not colocalized with either GABAergic or glutamatergic synapse markers remains unknown. It is conceivable that they represent extrasynaptic contact sites, either with the extracellular matrix or with non-neuronal cell types, e.g. with glial cells. In support of the latter notion, it was previously shown that a small population of oligodendrocytes express IgSF9b [17], giving rise to the possibility that IgSF9b may be involved in formation or maintenance of the myelin sheath. Consistent with this proposal, it was reported that in the axons of cultured hippocampal neurons, IgSF9b is absent from the non-myelinated axon initial segment but present in the myelinated distal axon [36]. Alternatively, it is possible that a subset of the observed IgSF9b puncta may represent intracellular storage sites that provide a reservoir of IgSF9b for plasticity processes.

Nevertheless, our study also confirms that IgSF9b does play a role in regulating the number and/or composition of a subset of GABAergic synapses in a brain region-dependent manner. Approximately 10–20% of GABAergic synapses in the brain regions investigated contain IgSF9b, and loss of IgSF9b expression results in a 10–20% reduction in gephyrin, Nlgn2, and/or VIAAT puncta in CA1 SLM, LHb, and MHb. Given these numbers, it is conceivable that deletion of IgSF9b leads to a near complete elimination specifically of the 10–20% of GABAergic synapses that do contain IgSF9b, while non-IgSF9b-containing synapses remain unaffected. GABAergic neurons are highly heterogenous population, and recent evidence indicates that different subtypes of GABAergic neurons utilize different complements of synaptic organizer proteins, creating a vast diversity of GABAergic synapses [15]. Prior evidence indicates that IgSF9b may function primarily at GABAergic synapses formed onto GABAergic inhibitory neurons, i.e. disinhibitory synapses [31]. This would be consistent with our prior observation that IgSF9b plays a particularly prominent role in the centromedial amygdala [32], a region which is comprised almost exclusively of GABAergic neurons, meaning that virtually all GABAergic synapses are disinhibitory synapses. Moreover, an analysis of IgSF9b expression patterns in different GABAergic neuron subtypes revealed highest expression levels in VIP-positive neurons [17], which form mostly disinhibitory synapses onto other GABAergic neurons. Together, these findings support the hypothesis that the fraction of IgSF9b that is present at GABAergic synapses may in part exert its influence on disinhibitory synapses.

The mechanism by which loss of function of IgSF9b affects GABAergic synapses remains largely unknown. While it was originally proposed that IgSF9b links to Nlgn2 via S-SCAM as a bridging molecule and thereby recruits gephyrin and other GABAergic synapse components [31], we subsequently found that, at least within the amygdala anxiety circuitry, IgSF9b and Nlgn2 do not appear to act at the same synapses [32]. Moreover, deletion of IgSF9b resulted in an increase in the number of VIAAT puncta in the CeM [32] as well as during development in deep layer 2/3 of visual cortex [33], arguing against a simple loss of GABAergic postsynaptic structures. IgSF9b is thought to form primarily homophilic interactions [31], and while this has yet to be confirmed in vivo, it indicates that its loss may differentially affect pre- and postsynaptic terminals. In support of this notion, a Drosophila homolog of the IgSF9 family, Borderless, was recently shown to regulate axonal transport of presynaptic components in a homophilic manner [37]. It is conceivable that IgSF9b may act by a similar mechanism to regulate the number or composition of VIAAT-containing presynaptic vesicles in addition to organizing postsynaptic protein complexes. However, why loss of IgSF9b appears to in some cases increase and in other cases decrease presynaptic VIAAT content remains unknown, as does the reason why changes in VIAAT are accompanied by postsynaptic alterations under some but not all conditions (Fig. 3 of our study, [32, 33]). Detailed molecular investigations will be needed to determine whether these changes occur differentially in a synapse subtype-dependent manner, and whether they are mediated directly by IgSF9b or are the result of homeostatic effects following other, as yet unidentified consequences of the loss of function of IgSF9b.

A further important aim of our study was to determine whether the neuroanatomical distribution of IgSF9b expression might explain its contribution to the etiology of neuropsychiatric disorders. The highest density of IgSF9b expression was identified in the stratum lacunosum moleculare of hippocampal area CA1, a region that contains the distal dendrites of CA1 pyramidal neurons and that receives dense glutamatergic inputs from the entorhinal cortex through the perforant pathway, as well as GABAergic inputs that mostly originate from oriens-lacunosum moleculare (OLM) interneurons and local neurogliaform cells. This region contributes to a wide range of aspects in memory formation and spatial context recognition, and it is possible that loss of function of IgSF9b may lead to disruptions in related cognitive functions, including those observed in patients with schizophrenia. To date, however, no direct analysis of the consequences of IgSF9b deletion on cognition, e.g. in IgSF9b KO mice, has been reported.

Prominent expression of IgSF9b was also observed in several regions that form part of or provide inputs to limbic circuits and are hence involved in the regulation of affective and depressive behaviors, including the lateral habenula [38–40], medial habenula [35, 41, 42], and basolateral amygdala [43–45]. These findings are particularly intriguing in light of the fact that IgSF9b was among the strongest loci identified in a GWAS meta-analysis of major depression [25]. Moreover, in a GWAS analysis of schizophrenia [21], it was also shown to be associated most pronouncedly with the negative symptoms of schizophrenia [20], which include flattened affect and emotional withdrawal. We had previously reported that global deletion of IgSF9b results in an anxiolytic effect that is particularly prominent in Nlgn2 KO mice, and that this effect could be partially mimicked by local virus-mediated deletion of IgSF9b in the centromedial amygdala [32]. These findings indicate that IgSF9b expression and function in multiple regions of the limbic system and associated brain areas may play an important role in regulating neuropsychiatrically relevant aspects of emotional behaviors.

Interestingly, high expression of IgSF9b was also observed in brain regions related to motor behaviors, including the globus pallidus, substantia nigra, and cerebellum, which may provide an explanation for its putative association with Parkinson’s disease [24, 26, 27]. Given that all of these regions contain prominent populations of GABAergic neurons, it is conceivable that these neurons may be particularly vulnerable to disruptions caused by variants in IgSF9b.

Taken together, our data confirm that the neuroanatomical expression pattern of IgSF9b is consistent with a role in the etiology of cognitive and affective disorders. Based on our findings and others, it is conceivable that IgSF9b variants may influence the function of psychiatrically relevant neuronal circuits either through effects at specific subsets of GABAergic synapses, particularly disinhibitory synapses, or through as yet unknown functions at glutamatergic excitatory synapses or additional neuronal contact sites. Future detailed analyses of the molecular, cellular and functional consequences of IgSF9b deletion at each of these sites will be essential to determine its contribution to human psychiatric disorders.

## Methods

### Animals

IgSF9b knockout mice were obtained from Lexington Pharmaceuticals (The Woodlands, TX, U.S.A.; Omnibank clone 281,214, generated through insertion of the Omnibank gene trap vector 48 into the IgSF9b gene in Sv129 ES cells) and were maintained on a C57BL/6 J background at the Max Planck Institute for Multidisciplinary Sciences as previously described [32]. For experiments involving comparison of WT and IgSF9b KO mice, IgSF9b heterozygous parents were crossed to generate WT and KO littermate offspring. For experiments involving only WT mice, WT littermates on a C57BL6/J background were used. Mice were group-housed (2–4 mice per cage) and were maintained on a 12 h light/dark cycle with food and water ad libitum. Only male mice between 8 and 14 weeks of age were used for experiments. Experimenters were blind to genotype during all stage of data acquisition and analysis. All procedures were conducted in accordance with the guidelines of the welfare of experimental animal use issued by the federal government of Germany, the Max Planck Society, and the Johannes Gutenberg University Mainz.

### Immunohistochemistry

Mice were anesthetized with isoflurane and immediately sacrificed for brain extraction. The brains were extracted and immediately immersed in 2-methylbutane at −35 °C for roughly 30 s. Brains were kept in the cryostat chamber at −18 °C for 30 min to allow the brains to acclimate, after which brain sections were cut at 18 μm thickness and mounted on glass slides. IgSF9b WT and KO samples were mounted on the same slides to ensure identical staining conditions between genotypes and experimental conditions. Subsequently, brain sections were dried at room temperature (RT) for at least 30 min. They were then fixed by one of two fixation methods, i.e. methanol fixation or paraformaldehyde (PFA) post-fixation, depending on the primary antibodies used as specified below. (1) For methanol fixation, brain sections were fixed in methanol at −18 °C for 5 min. (2) For PFA post-fixation, sections were fixed in 4% PFA (Carl Roth, Art. No. 2213.1) in phosphate buffer at RT for 5 min, followed by epitope retrieval in pre-heated citrate buffer (citric acid 0.1 M in dH_2_O, pH 6, Carl Roth Art. No. X863.1) at 100 °C for 30 min and subsequent cooling in the same solution at RT for 15 min. After fixation, slices were washed 3 × 10 min in 1X PBS and incubated for 1 h at RT with blocking solution (10% normal goat serum and 0.3% Triton X-100 in 1X PBS). They were then incubated overnight at 4 °C with primary antibodies (see below) diluted in blocking solution. On the next day, sections were washed 3 × 10 min in 1X PBS, incubated for 2 h at RT with secondary antibodies in blocking solution (see below), and again washed 3 × 10 min in 1X PBS, Finally, sections were incubated at RT for 10 min with DAPI (1:1000) (Invitrogen Art. No. 10184322) in blocking solution, washed 3 × 10 min in 1X PBS, and cover-slipped with ROTI Mount FluorCare (Carl Roth, No. HP19.1). For methanol-fixed sections, images were taken within 2–3 weeks after the fixation.

The following antibodies were used: (1) Primary antibodies used with methanol fixation: Guinea pig anti-Nlgn2 (RRID:AB_2619815, Synaptic Systems 129205, 1:500), mouse anti-Gephyrin (RRID:AB_2619837, Synaptic Systems 147111, 1:500), rabbit anti-IgSF9b (Atlas Antibodies Cat# HPA010802, RRID:AB_1079194, 1:200; 0.12 µg/µl, only for colocalization with Nlgn2 and gephyrin, Fig. 3). (2) Primary antibodies used with PFA post-fixation: Rabbit anti-IgSF9b (Atlas Antibodies Cat# HPA010802, RRID:AB_1079194, 1:200; 0.12 µg/µl, all experiments except colocalization with Nlgn2 and gephyrin), mouse anti-VIAAT (RRID:AB_2619818, Synaptic Systems 131011, 1:1000), mouse anti-VGLUT1 (RRID:AB_2617091, Synaptic Systems 135511, 1:1000, mouse anti-PSD-95 (RRID:AB_2877189**,** NeuroMab 75–028-20, 1:1000). It should be noted that due to a change in the concentration of anti-IgSF9b antibody provided by the supplier relative to our previous study (0.12 µg/µl in the current study compared to 0.5–1 μg/μl in Babaev et al. 2018 [32]), the dilution of this antibody was adapted from 1:1000 in the previous study to 1:200 in the present study to obtain the same final working concentration. (3) Secondary antibodies (all at 1:600, all from Thermo Fisher): Goat anti-rabbit Alexa Fluor 647 (RRID:AB_2633282, #A-32733), goat anti-mouse Alexa Fluor 555 (RRID:AB_2535844, #A-21422), goat anti-guinea pig Alexa Fluor 488 (RRID:AB_2534117, #A-11073).

### Image acquisition and processing for neuroanatomical characterization of IgSF9b expression

Images were acquired using a Leica SP8 laser scanning confocal microscope (Leica microsystems, Germany), equipped with Photomultiplier tube (PMT) and HyD detectors. A 63 × oil immersion objective with a Zoom 4 × was used to obtain single-plane micrographs at 1024 × 1024 spatial resolution with 4 × frame averaging. Laser power was optimized within the dynamic range of detection. The depth of the image captured corresponded to the plane with the highest signal intensity within the range of detection. For whole brain pictures (Fig. 1), a tiled overview was acquired at 256 × 256 pixel resolution with a 2 × optical zoom. For comparative quantification of IgSF9b puncta across brain regions (Fig. 2), six single plane images per animal and brain region (CA1 SLM, SR, SP, PML, MHb, LHb, PVT, LDT, BLA, CeA, CP) were obtained from a single brain section (at Bregma −1.4 mm) in order to ensure comparability. Data from all six images were subsequently averaged to obtain one final value for each animal and brain region. All acquisition and quantification imaging parameters were kept constant for all images from each experimental replicate. Puncta analysis was conducted using the Fiji ImageJ Software [46]. The same threshold value was used for all brain regions analyzed in each experimental animal (*n* = 5). To determine the threshold value for each animal, background intensity for every image was manually measured and averaged across all images, and the threshold for all brain regions was then defined at 2.5 × average background intensity. After setting the threshold, binarized images were subjected to noise despeckle and watershed segmentation in Fiji. Images were then subjected to the “Analyze Particles” segmentation algorithm using a filter size of 0.03–1.03µm^2^ for IgSF9b puncta.

### Image acquisition and processing for quantification of inhibitory synapse markers in IgSF9b KO mice

Images were obtained using a Leica SP8 laser scanning confocal microscope (Leica microsystems, Germany), equipped with Photomultiplier tube (PMT) and HyD detectors. A 63 × oil immersion objective with a Zoom 4 × was used to obtain single plane micrographs at 1024 × 1024 spatial resolution with 4 × frame averaging. Laser power was optimized within the dynamic range of detection. For each WT-KO pair (*n* = 7) and brain region (CA1 SLM, MHb, LHb, BLA), 10 single plane images were acquired per condition. Within each pair of WT and IgSF9b KO mice and brain region comparison, imaging parameters were kept constant. Image analysis was conducted with the Fiji ImageJ software [46]. The same threshold was used in each experimental WT-KO pair (*n* = 7) within each brain region [46]. To determine the threshold value for each animal, background intensity for every image was manually measured and averaged across all images belonging to the same WT-KO pair and brain region, and the threshold for that WT-KO pair and brain region was defined at 2.5 × average background intensity. After setting the threshold, binarized images were subjected to noise despeckle and watershed segmentation in Fiji. Images were then subjected to the “Analyze Particles” segmentation algorithm using a filter size of 0.03–1 µm^2^ for gephyrin and Nlgn2 puncta and 0.03–3 µm^2^ for VIAAT puncta. Number and size of puncta were quantified for each image, normalized to the average value from all images within a WT-KO pair, and expressed as % WT control.

### Image acquisition for IgSF9b object-based colocalization analysis

Images were obtained using a Leica SP8 laser scanning confocal microscope (Leica microsystems, Germany), equipped with Photomultiplier tube (PMT) and HyD detectors. A 63 × oil immersion objective with a Zoom 4 × was used to obtain single-plane micrographs at 1024 × 1024 spatial resolution with 4 × frame averaging. Laser power was optimized within the dynamic range of detection. For each animal (*n* = 6–7) and brain region (CA1 SLM, MHb, LHb, BLA), 10 single plane images were acquired per condition. 2D single plane images were chosen rather than 3D z-stacks, since the methanol fixation protocol used for colocalization with gephyrin and Nlgn2 leads to a substantial reduction in section thickness, which precludes the acquisition of meaningful z-stacks.

Colocalization analysis was conducted with the Fiji ImageJ software [46]. To determine the threshold value for each animal, background intensity for every image was manually measured and averaged across all images belonging to the same brain region, and the threshold for that brain region was then defined at 2.5 × background intensity. After setting the threshold, binarized images were subjected to noise despeckle and watershed segmentation in Fiji. Images were then subjected to the “Analyze Particles” segmentation algorithm using a filter size of 0.03–1 µm^2^ for IgSF9b, Nlgn2, gephyrin, and PSD-95, and 0.03–3 µm^2^ for VIAAT and VGLUT1. Subsequently, a “Count Mask” image was generated of each marker and subjected to object-based colocalization analysis using the ImageJ Plugin JACoP [47] to colocalize IgSF9b puncta with other synaptic markers. Colocalization puncta were expressed as percentage of total synapse marker puncta (to quantify the percentage of synapse puncta that contain IgSF9b, Fig. 4h-k and Fig. 5h-k) or as percentage of total IgSF9b puncta (to quantify the percentage of IgSF9b puncta that are localized at synapses, Fig. 4l-o and Fig. 5l-o). Examples of colocalized puncta that were included in the analysis, as well as apposed puncta that were excluded from this analysis, can be found in Figure S3. Pearson correlation analysis (Figure S4) was performed using the same count mask as for the above colocalization analysis.

### Statistical analysis

Statistical analysis for the comparison of data from WT and IgSF9b KO mice was performed using Prism (GraphPad Software, La Jolla, CA, USA). Normality of data sets was assessed using the Shapiro–Wilk test and data from WT and IgSF9b KO mice were subjected an unpaired, two-tailed Student’s t-test or a Mann–Whitney-U test for normally or non-normally distributed data sets, respectively, with the IgSF9b WT/KO genotype as comparing factors. All statistical analysis data can be found in Table S1.

## Supplementary Information

Below is the link to the electronic supplementary material.

Supplementary file1 (PDF 1592 KB)

## Data Availability

All the programs used to analyze the images and generate the data are freely available on https://imagej.net/software/fiji/downloads.
