# Supplementary material for: Neuroanatomical characterization of the cell adhesion molecule IgSF9b reveals localization to inhibitory and excitatory synapses in the mouse limbic system
Source: J Mol Med (Berl). 2025 Oct 17;103(11-12):1551–65. doi: 10.1007/s00109-025-02600-6 (PMC12675716; doi:10.1007/s00109-025-02600-6)
Supplement: Supplementary file 1 — Supplementary file1 (PDF 1592 KB) [file 109_2025_2600_MOESM1_ESM.pdf]

SUPPLEMENTARY INFORMATION

Neuroanatomical characterization of the cell adhesion molecule IgSF9b reveals localization to inhibitory and excitatory synapses in the mouse limbic system.

Federico Rotondo, Heba Ali, Maxim Maichle,
Michael J. Schmeisser, Nils Brose, and Dilja Krueger-Burg

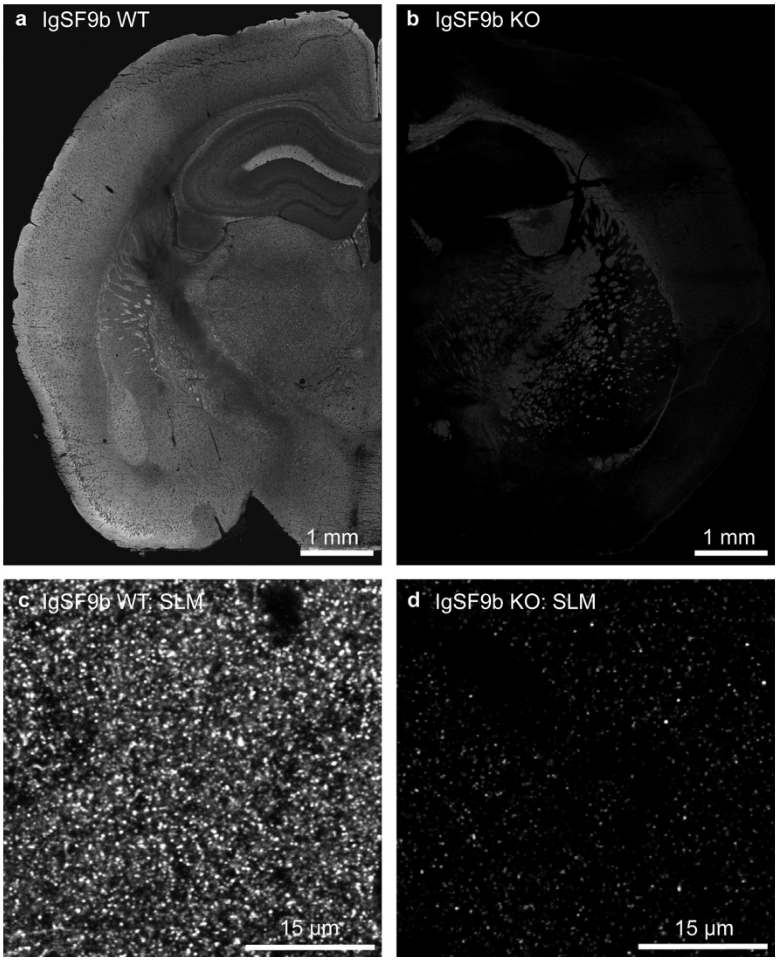

Figure S1. Validation of the IgSF9b antibody. **a-b** Photomicrographs showing immunostaining for IgSF9b in a coronal brain section in a WT (a) and IgSF9b KO (b) mouse. **c-d** High magnification photomicrographs showing immunostaining for IgSF9b in the stratum lacunosum moleculare (SLM) of hippocampal area CA1 of a WT (c) and IgSF9b KO (d) mouse.

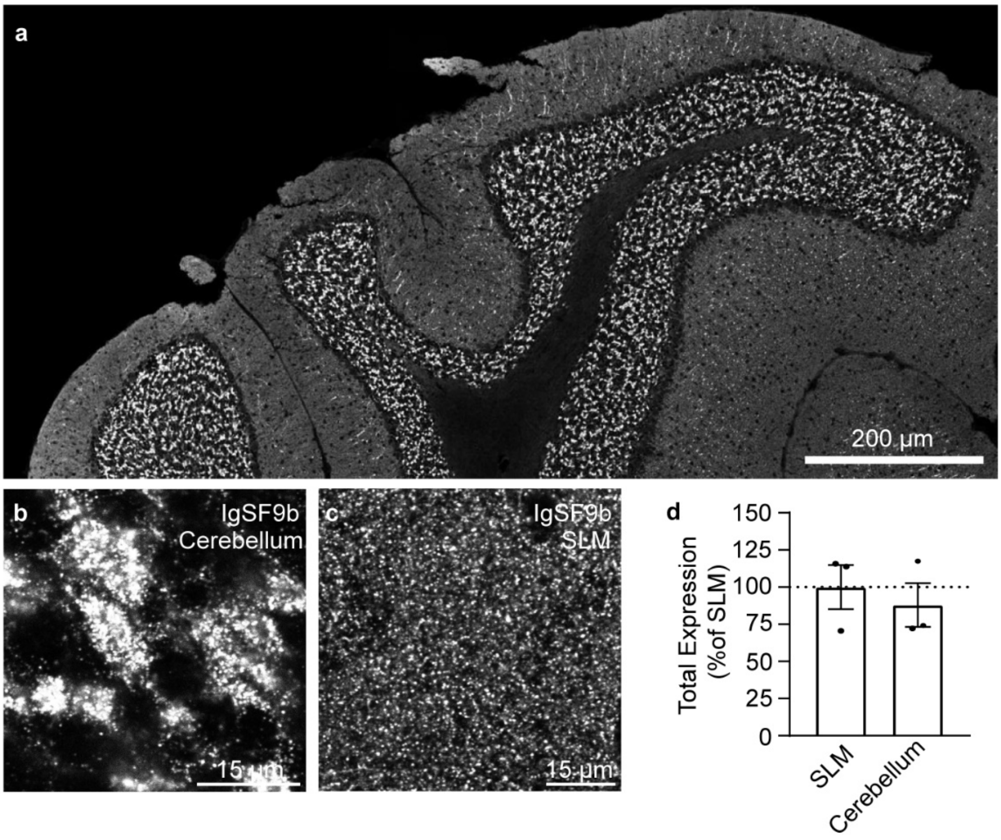

Figure S2. Quantification of IgSF9b in the granular layer of the cerebellum. **a** Overview of IgSF9b staining in the cerebellum **b-c** Comparison of IgSF9b immunostaining in the granular layer of the cerebellum and the stratum lacunosum moleculare (SLM) of hippocampal area CA1, with representative photomicrographs of IgSF9b immunostaining in the granular layer of the cerebellum (b) and the SLM of hippocampal area CA1 (c). **d** Quantification of the total expression (number of puncta x size of puncta) of IgSF9b in both regions, normalized to the total expression of IgSF9b in the SLM for comparison.

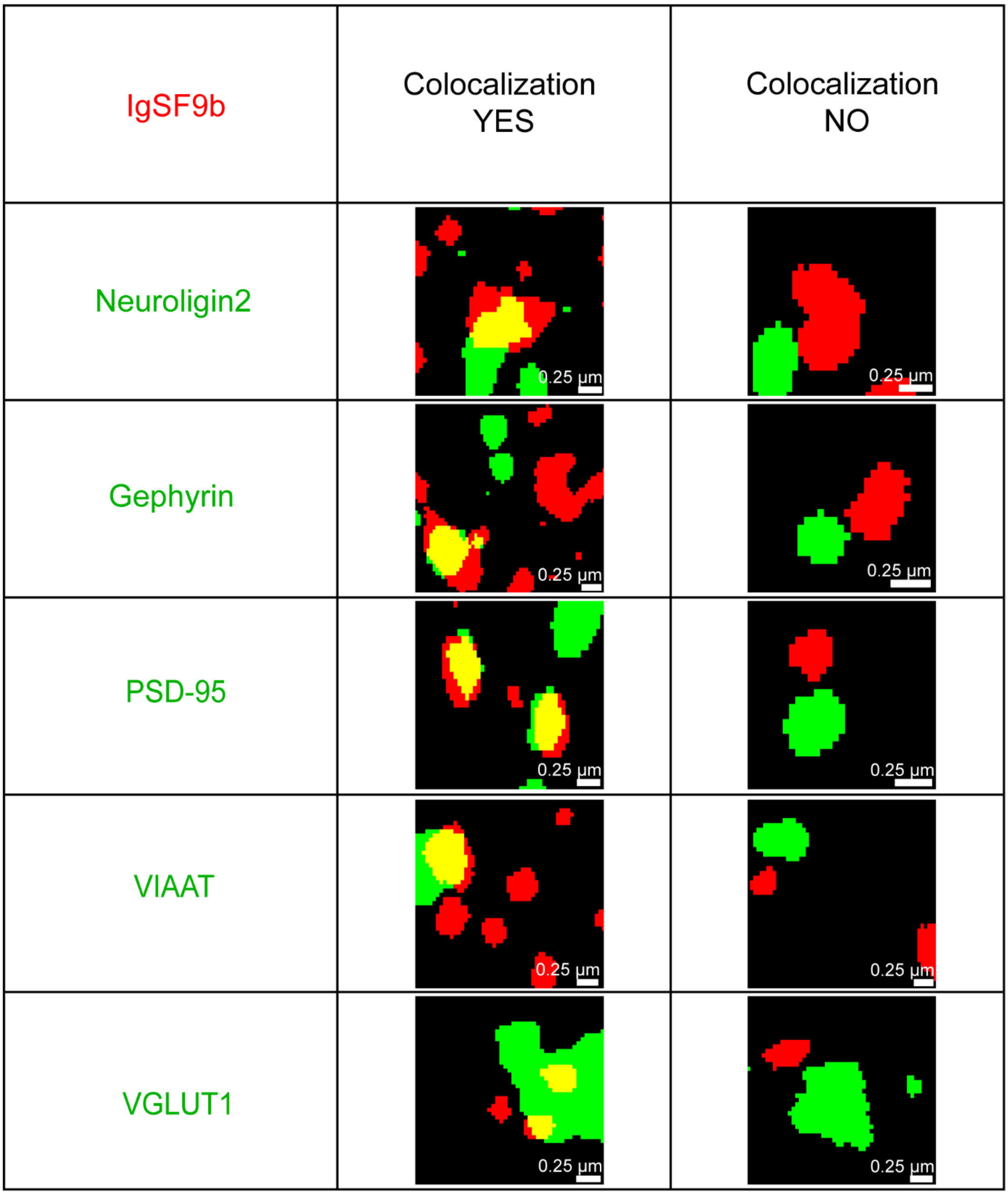

Figure S3. Example images for the colocalization analysis of IgSF9b with inhibitory and excitatory synapse markers. For each marker, the left image shows an example of fully colocalized puncta that were included in the quantification in figures 4 and 5, while the right image shows puncta that are adjacent but that were not counted as colocalized in the quantification in figures 4 and 5.

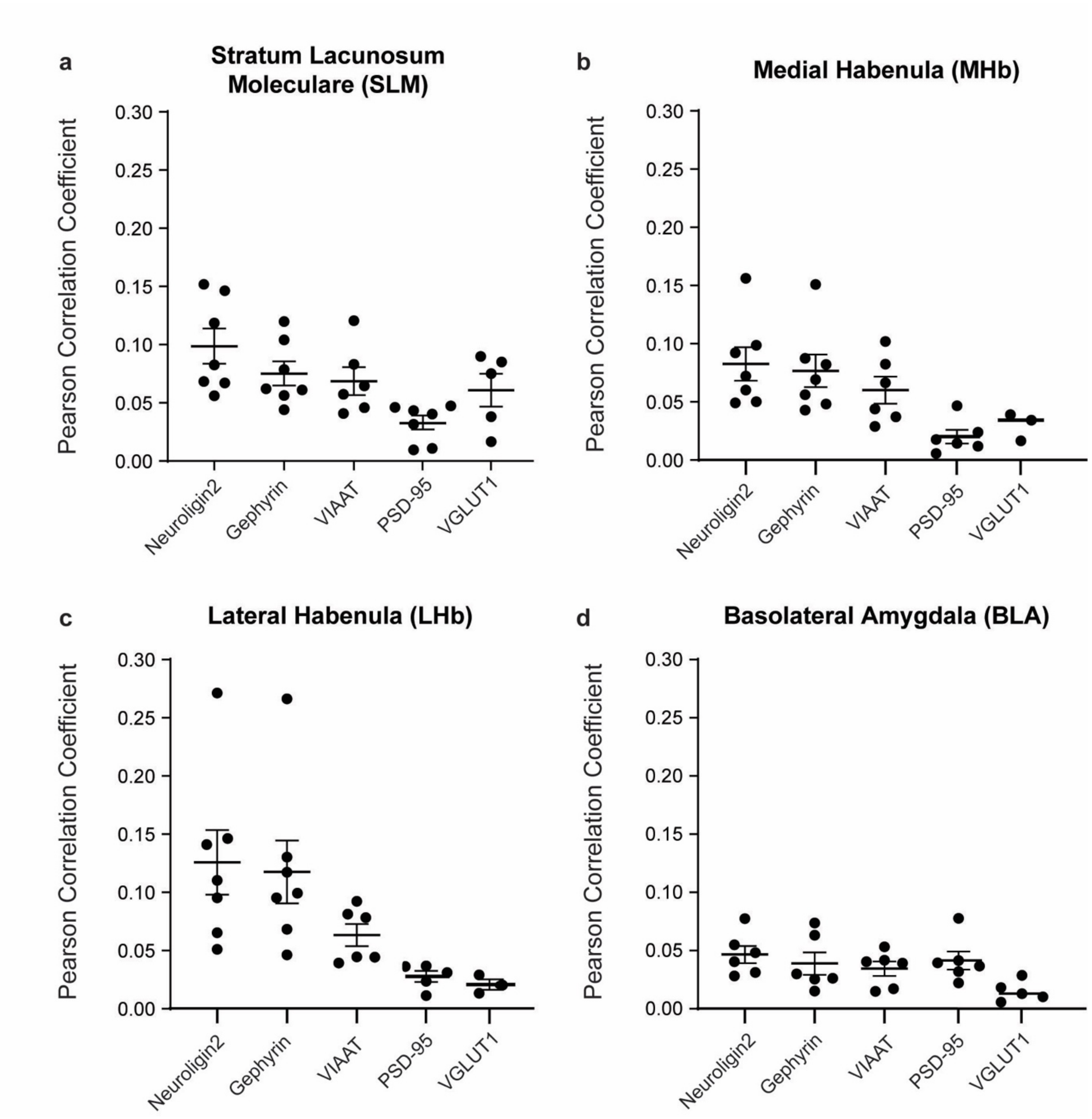

Figure S4. Pearson correlation coefficient for colocalization data shown in Figures 4 and 5. For each image analysed, the Pearson correlation coefficient between IgSF9b puncta and the respective synaptic marker puncta was determined using the corresponding Fiji ImageJ software function, and the average Pearson correlation coefficient was subsequently calculated for each animal and brain region.

Table S1. Statistical data from the comparison of inhibitory synapse markers in WT and IgSF9b KO mice (Figure 3).

	IgSF9b WT-KO	Normally Distributed	P-Value	Significance
SLM	Number of Gephyrin Puncta	Yes	0.040	*
	Size of Gephyrin Puncta	Yes	0.263	ns
	Number of Nlgn2 Puncta	Yes	0.112	ns
	Size of Nlgn2 Puncta	Yes	0.019	*
	Number of VIAAT Puncta	Yes	0.015	*
	Size of VIAAT Puncta	Yes	0.001	**
MHb	Number of Gephyrin Puncta	Yes	0.750	ns
	Size of Gephyrin Puncta	Yes	0.061	ns
	Number of Nlgn2 Puncta	No	0.545	ns
	Size of Nlgn2 Puncta	Yes	0.024	*
	Number of VIAAT Puncta	Yes	0.001	**
	Size of VIAAT Puncta	Yes	0.079	ns
LHb	Number of Gephyrin Puncta	Yes	0.562	ns
	Size of Gephyrin Puncta	No	<0.0001	****
	Number of Nlgn2 Puncta	Yes	0.496	ns
	Size of Nlgn2 Puncta	Yes	0.0004	***
	Number of VIAAT Puncta	Yes	0.029	*
	Size of VIAAT Puncta	Yes	0.806	ns
BLA	Number of Gephyrin Puncta	Yes	0.615	ns
	Size of Gephyrin Puncta	Yes	0.254	ns
	Number of Nlgn2 Puncta	Yes	0.518	ns
	Size of Nlgn2 Puncta	Yes	0.203	ns
	Number of VIAAT Puncta	Yes	0.172	ns
	Size of VIAAT Puncta	Yes	0.439	ns
